# Knowledge and Awareness of Osteoporosis: A Survey of Physical Therapy Providers in Saudi Arabia

**DOI:** 10.1155/2024/2797382

**Published:** 2024-03-18

**Authors:** Muataz Almaddah, Fahad Alzahrani, RiziqAllah Gaowgzeh, Abdullah Alqarni, Rani Othman, Afnan Gmmash

**Affiliations:** Department of Physical Therapy, Faculty of Medical Rehabilitation Sciences, King Abdulaziz University, Jeddah, Saudi Arabia

## Abstract

**Background:**

Osteoporosis “OP” is classified as one of the most serious health conditions worldwide. OP increases the skeletal fracture risk by 35%, particularly at hip, spine, and wrist joints. Healthcare professionals should be aware of OP clinical signs and have good knowledge while managing all patients.

**Objectives:**

This study aims to investigate the current level of osteoporosis knowledge and awareness among physical therapy providers in Saudi Arabia.

**Methods:**

One hundred and sixty-eight physical therapy providers participated in this cross-sectional electronic survey from February to July of 2021. The participants completed the Osteoporosis Knowledge Assessment Tool questionnaire (OKAT). Descriptive analysis was utilized to assess the current level of osteoporosis knowledge among physical therapy providers.

**Results:**

Among the 168 participants, 55% (*n* = 92) were over 31 years old and 45% (*n* = 76) were 30 years old or under. The majority of participants 37% (*n* = 62) had more than 10 years of experience, 45% (*n* = 76) mainly treat orthopedic conditions, and 70% (*n* = 117) live in the western region. The results showed that 67.9% (*n* = 114) of participants had good knowledge about osteoporosis, while 19.6% (*n* = 33) had poor knowledge, and only 12.5% (*n* = 21) had excellent knowledge.

**Conclusion:**

Physical therapy providers in Saudi Arabia have a good knowledge of osteoporosis. The overall OP preventive measure knowledge questions were poor. It is crucial for physical therapy providers to act appropriately to prevent falls and mitigate any potential risks.

## 1. Introduction

Osteoporosis (OP) is classified as one of the most serious health conditions [1]. OP is the second most common disease in developed countries following heart diseases [2]. OP affects 33% of women and 20% of men over the age of 50 years, resulting in serious complications such as skeletal fractures, disability, decreased quality of life, and an increased mortality rate (15–30%) similar to breast cancer and cerebrovascular accidents [3–5].

OP is typically asymptomatic, especially in the early stages, until skeletal fracture occur [6]. Several major risk factors for OP have been reported in the literature, including genetics, poor eating habits, and sedentary lifestyles [7]. In addition, people suffering from comorbidity diseases, such as chronic obstructive pulmonary disease, malignancy, type 2 diabetes mellitus, obesity, liver diseases, and atherosclerosis, are at risk of developing OP [8].

In the Saudi population particularly, the prevalence of OP has been found to be higher than in western countries [9]. Osteoporosis affects 30% of all postmenopausal women in the USA and Europe [10, 11]. In Saudi Arabia, recent studies found that OP affects 53% of Saudi women [12]. OP appears to occur at a younger age in the Saudi population when compared to the western counterparts [13]. OP-related fractures are expected to impact 6 million people globally by 2050 [5, 14]. In particular, hip fractures affect one in every 1000 women over the age of 50 years [15]. Furthermore, by 2025, the cost of hip fracture health care is predicted to exceed 35 billion Saudi riyal. Based on the existing data, it is anticipated that more than half a million OP fractures will occur in Saudi Arabia over the next three years if no rigorous actions are taken [16].

Therefore, prioritizing the early detection of osteoporosis in patients and preventing fractures are essential for physical therapy providers to ensure safe and timely intervention sessions without posing any harm to vulnerable populations [17, 18]. In addition, the level of knowledge of healthcare providers including physical therapists plays an integral role in early detection and prevention of OP, thereby significantly enhancing patient care [16, 17].

Several studies have assessed the level of knowledge for OP among Saudi physicians and other healthcare providers. Almalki et al. conducted a cross-sectional study where 140 interns participated from nine medical schools [19]. The study found poor OP-related knowledge among the medical interns. Similarly, inadequate knowledge of OP was found among 75 general medical practitioners and 120 nurses in another study [20].

Moreover, Alghamdi and Mohammed conducted a cross-sectional study at Bisha hospital including 141 health professionals (medical, surgical, primary health care, and nursing departments) to assess their knowledge and awareness of OP [21]. This study found that health professionals have a strong understanding of osteoporosis, but that there is no significant difference in osteoporosis knowledge between the different subgroups of health professionals (age groups, sex, experience, and specialty). Similarly, there was a considerable level of knowledge and awareness towards understanding OP among different healthcare professionals working in Al Majma'ah city' hospitals [22].

Although previous studies investigated OP knowledge among health professionals such as physicians, nurses, and other healthcare providers, none explored the level of OP knowledge and awareness among physical therapy providers. Investigating physical therapy providers' knowledge and awareness related to OP would highlight the need to promote their practice capacity, which allows cost-effective treatment and reduces the occurrence of osteoporotic fractures in low to high-risk patients. Therefore, this study investigated the current level of osteoporosis knowledge and awareness among physical therapy providers in Saudi Arabia.

## 2. Methods

### 2.1. Study Design

A cross-sectional survey among the physical therapy providers in Saudi Arabia was conducted from February 2021 to July 2021. The survey aimed to assess the level of knowledge and awareness towards OP along with its risk factors.

### 2.2. Survey

The Google forms platform was used for data collection. The questionnaire was created in English and distributed via social communication platforms, physical therapists e-mail address lists, word of mouth, and contacting physical therapy providers directly. The survey information sheet explained the aim of the present study. Only electronic consent forms were taken from all participants. The demographic data such as age, gender, place of work, region, years of experience, college, qualifications, nationality, and area of physical therapy specialty were obtained from all the participants.

### 2.3. Sampling

Sampling included physical therapists in Saudi Arabia currently working at hospitals or clinics, either governmental or private. A priori sample size calculation was conducted to estimate the number of respondents required in the study to provide meaningful estimates of percentages. The number of physical therapists in Saudi Arabia is approximately 4000 physical therapists [23]. G-power was utilized to estimate the sample size required to have a power of 80%. Using a medium effect size of 0.3 and a margin error of 0.05, the sample size required was 350 physical therapists.

### 2.4. Assessment Tool

One of the most OP common tools is the Osteoporosis Knowledge Assessment Tool (OKAT) which has been used in the present study. The major advantages of OKAT are simple to deliver and do not take more than 5 minutes to fill the form. It is a valid and reliable questionnaire to assess knowledge about OP. The OKAT is a good measure of OP knowledge and was developed by Winzenberg et al. [24].

The OKAT questionnaire is composed of 20 items to assess the participant's knowledge related to OP. All questions have to be answered as “true,” “false,” or “do not know.” The items of the OKAT were scored as follows: One point is given for true while zero for false and for “do not know” answers. The total score of participants' answers was based on the conceptual framework proposed by Alqahtani and Alghamdi [7], where a total score of 0–7 was classified as poor knowledge, a total score of 8–13 was considered having good knowledge, and a score of 14 points or higher was considered as posing excellent knowledge about OP.

### 2.5. Statistical Analysis

Descriptive analysis generated to express data frequency and percentage of the participants' demographic information and OKAT responses. Data were managed and analysed using Statistical Package for Social Sciences (SPSS) version 26 (IBM Corporation, Armonk, NY). Normality was assessed using the Kolmogorov–Smirnov test. Subanalysis was performed utilizing the Pearson chi-square to test for association between different variables and OP level of knowledge. *P* values of <0.05 were considered significant.

### 2.6. Ethics

The Ethical Review Board of the Faculty of Medical Rehabilitation Sciences on February 11^th^, 2021, has reviewed and approved the study before any data collection (IRB Number: FMRS-EC202-04).

## 3. Results

The total number of respondents was *n* = 224, and respondents who completed the entire survey and included in the analysis were *n* = 168. Among the 168 respondents, 72% (*n* = 121) were males and 96.4% (*n* = 162) were Saudi nationals. Regarding the education qualification, 74% (*n* = 124) of the respondents had a bachelor's degree (Table 1).

There were responders from different age groups, with 50% (*n* = 84) of the participants between the age of 31 and 40 years. Regarding the region of practice in Saudi Arabia, most of the participants 70% (*n* = 117) work in the western region and the rest work in the rest of four main regions of Saudi Arabia. Around 38.7% of the participants pursued their bachelor's degree at Taif University, and 13.7% studied at Umm Al-Qura University, followed by King Abdul Aziz University with 10.1%.

Regarding the years of experience, 36.9% of the participants have more than ten years of experience, while 28% have experience between one and five years. Finally, most of the providers (45.2%) mainly work with orthopedic cases, and the rest of the participants work with pediatrics, geriatric, sports injuries, and long-term care units.

After the frequencies and percentages of the personal, scientific, and practical information were drawn, the twenty questions of OKAT are analysed and presented in Table 2.

Most of the participants (94.6%) got the correct response for the OKAT1 (OP leads to an increased risk of bone fractures). This is followed by the item OKAT4 (OP is more common in men) with 82.7% of the participants answered correctly. On the other side, 77.4% of the participants had the wrong answer for the items OKAT18 (there is a small amount of bone loss in the ten years following the onset of menopause), followed by the question OKAT2 with a 57.6% as a percentage of wrong answers.

The normality test shows that skewness was −0.019 with a standard error of 0.187, and the kurtosis was 0.146 with a standard error of 0.373. The values of skewness and kurtosis were within the threshold of −/+2, and the normal distribution was verified.

Pearson chi-square analysis results are shown in Table 3 for all variables in association to OP knowledge levels. The participants' gender has no association with OP knowledge, with *P* value = (0.331). Participants who completed their postgraduate studies had the highest knowledge about osteoporosis followed by those with a bachelor's degree. There were no association between the OP knowledge and the respondents' physical therapy qualification *P* value (0.55).

The highest level of OP knowledge was recorded for participants within the age group of 31–40 years and respondents' working in the southern region. The years of experience groups did not show any association with OP knowledge. Finally, the differences between the conditions that they mainly manage showed a significant association with OP knowledge with a *P* value of (0.028).

## 4. Discussion

Osteoporosis is a public health problem characterized by loss of bone mineral density. Numerous studies conducted in Saudi Arabia have consistently demonstrated that the prevalence of osteoporosis is significantly higher than in the US and Europe [25]. It is important to note that vitamin D deficiency is extremely common among Saudis in general, affecting more women than men as well as kids and teenagers. It is essential to improve knowledge and awareness among healthcare providers, especially physical therapy providers in context to osteoporosis. Physical therapy practice involves patient gait training, and this might put some patients at a higher risk of fall. Therefore, it is relatively the first study to investigate the current knowledge about osteoporosis among physical therapy providers working in Saudi Arabia by adopting OKAT as an instrument for assessing awareness on osteoporosis. While planning this study, it was hypothesized that there is adequate knowledge of osteoporosis among physical therapists working in Saudi Arabia.

One hundred and sixty-eight valid responses were analysed and included in the study. The majority of participants were males (72%), holding a bachelor degree in physical therapy (73.8%). The study initially targeted the physical therapist at a national level; however, the majority of the respondents were practicing in Makkah region (67%), and this is due to the fact the study researchers were located in Makkah region and the most of their connections within the same region. Despite the centred response area, we received response from the majority of Saudi Arabia regions.

In the current study, 68 percent of the participants expressed good knowledge of osteoporosis, which reflects satisfactory result, and 12.5% demonstrated excellent knowledge. This finding can be explained by the rise in the awareness campaign done annually among medical providers in Saudi Arabia including physical therapists [26]. Another reason for seeing a good OP knowledge among the participants is the mandatory national accreditation for physical therapy programs in Saudi Arabia while benchmarking the program curriculum with the lead programs in physical therapy internationally. The current findings are in agreement with another study carried out in Saudi Arabia assessing the healthcare providers' awareness when they found a good knowledge in 90% of the participants [21]. Conversely, another study found poor osteoporosis knowledge (60%) among medical interns [19]. A review study by Nguyen concluded that healthcare providers lack OP awareness [27].

Concerning the osteoporosis OKAT score, the majority of respondents demonstrated a good level of knowledge; however, a large gap in knowledge of osteoporosis was detected in three questions assessing OP symptoms (salt intake and menopause relation to osteoporosis). The possible reason for the limited knowledge related to those three questions could be due to the fact that male physical therapists typically do not treat female cases in general, and the majority of the participants were males (74%). On the effect of gender, the present study showed higher knowledge scores to be associated with males, and this could be explained by the difference between the number of participants from each gender.

There were no statistically significant differences in the degree of osteoporosis knowledge between age groups, gender, or length of experience in our findings, despite previous studies demonstrating a substantial inverse relationship between physicians, age, and awareness of osteoporosis [19–21]. This discrepancy might be due to the small sample size in the current study but could also be linked to the heterogeneity of our study population. Furthermore, Alqahtani and Alghamdi recently documented poor knowledge among young participants [7]. Moreover, the same study found no difference in knowledge among male and female genders regarding osteoporosis [7]. Ahmed et al. found knowledge about osteoporosis did not differ between age groups, and the same was concluded by other studies [22, 28, 29]. The means of the different education groups, on the other hand, were considerably different. It is commonly known that education level influences the knowledge level, and this finding was verified by Werner et al.; yet, researchers such as Riaz et al. discovered the contrary of our findings [2, 30].

The present study points out the knowledge of osteoporosis among physical therapy providers. As a result, these findings emphasize the importance of physical therapists in Saudi Arabia expanding their knowledge of screening patients at risk of osteoporosis and practicing osteoporosis prevention. In addition, therapists should incorporate physical activity in collaboration with high impact exercise, aerobic exercise, daily working strengthening and resisted exercises, and balance training to prevent falls and their subsequent complications.

This study also found that physical therapists who work with senior patients have a high level of knowledge. This was expected, given geriatric physical therapists are trained in diagnosing and addressing numerous age-related diseases, particularly OP.

**Figure 1:**
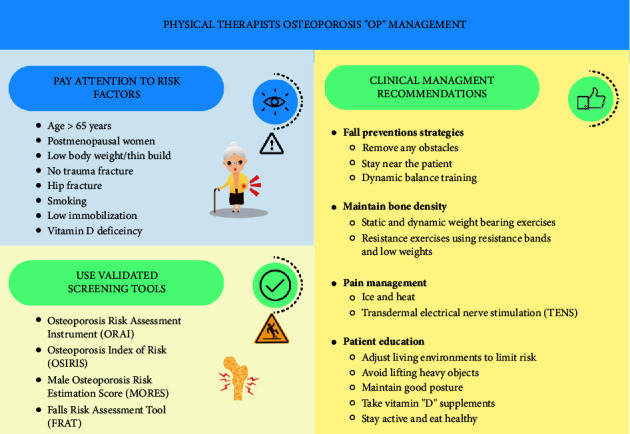
Summary tool for physical therapists on how to assess and manage osteoporosis patients.

Physical therapists should update their knowledge more frequently especially with those at risk of having OP. In clinical settings, attention should be paid to risk factors when taking history (women, above 65 years, history of fracture, and vitamin D deficiency). During treatment session, we should plan for a safe environment and mind the contraindications and precautions. During the initial assessment, we should consider using the Osteoporosis Risk Assessment Instrument (ORAI) or Osteoporosis Index of Risk (OSIRIS). These tools seem to have a similarity and are moderately accurate at predicting osteoporosis. We have created a summary tool for physical therapists to detect osteoporotic patients and minimize their risk of fall and secondary complications (Figure 1) [31–33]. The summary tool intended to facilitate decision-making by physical therapists treating individuals with known or suspected osteoporosis.

### 4.1. Limitations

The cross-sectional design and convenience sample utilized in the current study were limitations of this study. Furthermore, the majority of respondents in the study were from the western region, which reflect their OP knowledge and awareness. Therefore, a larger sample size from all Saudi Arabia regions is recommended to ensure generalizability of the study results.

### 4.2. Recommendation

Physical therapists should be encouraged to participate in programs of continuing medical education and short-term courses that allow them to refresh their knowledge and treatment plan in relation to the most recent evidence from time to time.

## 5. Conclusion

This study concludes that physical therapists in Saudi Arabia have a great knowledge of osteoporosis. However, scores were relatively low in items of practice and prevention of osteoporosis making it a concerning indicator and creating modes to develop policies concentrating on prevention of OP. In addition, participants with a postgraduate degree have a higher level of knowledge. Therefore, physical therapists are encouraged to gain and accelerate their expertise while also attending education programs and postgraduate courses for greater understanding and the development of new conceptual criteria for OP.

## Figures and Tables

**Table 1:** Demographic characteristics of the participants.

	Total participants = 168	*n* (%)
Gender	Male	121 (72)
	Female	47 (28)

Age	21–30 years	76 (45)
	31–40 years	84 (50)
	41–50 years	8 (5)

Qualification	Diploma degree	13 (7.5)
	Bachelor's degree	124 (74)
	Postgraduate degree	31 (18.5)

Region of practice	Central	25 (15)
	Western	117 (70)
	North	8 (4.5)
	South	12 (7)
	Eastern	6 (3.5)

Education	King AbdulAziz University	17 (10)
	King Saud University	12 (7)
	Taif University	65 (39)
	Umm Al-Qura University	23 (14)
	Other	51 (30)

Experience	Less than 1 year	26 (15.5)
	1–5 years	47 (28)
	5–10 years	33 (19.5)
	More than 10 years	62 (37)

Speciality	Pediatrics	18 (11)
	Geriatrics	14 (8)
	Sport injuries	15 (9)
	Long-term care units	11 (6.5)
	Orthopedics	76 (45)
	Others	34 (20.5)

**Table 2:** Frequency and percentages of responses and answer accuracy of OKAT.

^*∗*^OKAT items	Responses (*n* = 168)			Wrong answer (%)	Correct answer (%)
	False	True	^ǂ^DK		
OKAT1: osteoporosis leads to an increased risk of bone fractures	6 (3.6%)	**159 (94.6%)**	3 (1.8%)	5.40	94.6
OKAT2: osteoporosis usually causes symptoms (e.g. pain) before fractures occur	**41 (24.4%)**	108 (64.3%)	19 (11.3%)	75.6	24.4
OKAT3: having a higher peak bone mass at the end of childhood gives no protection against the development of osteoporosis in later life	**55 (32.8%)**	56 (33.3%)	57 (33.9%)	66.7	33.3
OKAT4: osteoporosis is more common in men	**139 (82.7%)**	17 (10.1%)	12 (7.2%)	17.3	82.7
OKAT5: cigarette smoking can contribute to osteoporosis	12 (7.2%)	**120 (71.4%)**	36 (21.4%)	28.6	71.4
OKAT6: white women are at highest risk of fracture as compared to other races	20 (11.9%)	**81 (48.2%)**	67 (39.9%)	51.8	48.2
OKAT7: a fall is just as important as low bone strength in causing fractures	29 (17.2%)	**110 (65.6%)**	29 (17.2%)	34.4	65.6
OKAT8: by age 80, the majority of women have osteoporosis	15 (8.9%)	**133 (79.2%)**	20 (11.9%)	20.8	79.2
OKAT9: from age 50, most women can expect at least one fracture before they die	44 (26.2%)	**64 (38.1%)**	60 (35.7%)	61.9	38.1
OKAT10: any type of physical activity is beneficial for osteoporosis	**79 (47%)**	61 (36.3%)	28 (16.7%)	53	47
OKAT11: it is easy to tell whether I am at risk of osteoporosis by my clinical risk factors	34 (20.2%)	**86 (51.2%)**	48 (28.6%)	48.8	51.2
OKAT12: family history of osteoporosis strongly predisposes a person to osteoporosis	30 (17.9%)	**104 (61.9%)**	34 (20.2%)	38.1	61.9
OKAT13: an adequate calcium intake can be achieved from two glasses of milk a day	24 (14.3%)	**75 (44.6%)**	69 (41.1%)	55.4	44.6
OKAT14: sardines and broccoli are good sources of calcium for people who cannot take dairy products	16 (9.5%)	**82 (48.8%)**	70 (41.7%)	51.2	48.8
OKAT15: calcium supplements alone can prevent bone loss	**104 (61.9%)**	34 (20.2%)	30 (17.9%)	38.1	61.9
OKAT16: alcohol in moderation has little effect on osteoporosis	69 (41.1%)	**43 (25.6%)**	56 (33.3%)	74.4	25.6
OKAT17: a high salt intake is a risk factor for osteoporosis	37 (22%)	**42 (25%)**	89 (53%)	75.0	25.0
OKAT18: there is a small amount of bone loss in the ten years following the onset of menopause	**38 (22.6%)**	75 (44.7%)	55 (32.7%)	77.4	22.6
OKAT19: hormone therapy prevents further bone loss at any age after menopause	27 (16.1%)	**69 (41.1%)**	72 (42.9%)	58.9	41.1
OKAT20: there are no effective treatments for osteoporosis available in KSA	**92 (54.8%)**	30 (17.9%)	46 (27.4%)	45.2	54.8

^*∗*^OKAT = Osteoporosis Knowledge Assessment Tool. ^ǂ^DK **=** do not know. Bold = the correct answer to OKAT items.

**Table 3:** Pearson chi-square analysis comparing different characteristics of the participants and osteoporosis knowledge categories.

	Total = 168	^*∗*^OKAT knowledge categories				OKAT score/20
	Variable (*n*)	Poor^ƚ^*n* (%)	Good *n* (%)	Excellent *n* (%)	Pearson *P* value	Mean ± SD
Gender	Male (121)	34 (20%)	85 (50.6%)	2 (1.2%)	0.331	9 ± 3
	Female (47)	9 (5.5%)	36 (21.5%)	2 (1.2%)		9.6 ± 2

Age	21–30 years (76)	20 (12%)	54 (32%)	2 (1.2%)	0.992	9 ± 2.3
	31–40 years (84)	21 (12.8%)	61 (36%)	2 (1.2%)		9.2 ± 3
	41–50 years (8)	2 (1.2%)	6 (3.6%)	0		10.8 ± 3.5

Qualification	Diploma degree (13)	4 (2.4%)	9 (5.5%)	0	0.55	8.7 ± 2.7
	Bachelor's degree (124)	32 (19%)	90 (53.6%)	2 (1.2%)		9 ± 2.6
	Postgraduate degree (31)	7 (4.2%)	22 (13%)	2 (1.2%)		10.5 ± 3

Region of practice	Central (25)	3 (1.8%)	21 (12.8%)	1 (0.6%)	0.407	10 ± 1.6
	Western (117)	35 (20.9%)	80 (47.6%)	2 (1.2%)		9 ± 3
	North (8)	6 (3.6%)	2 (1.2%)	0		11 ± 0.5
	South (12)	2 (1.2%)	10 (6%)	0		11.5 ± 2
	Eastern (6)	2 (1.2%)	4 (2.4%)	2 (1.2%)		10.3 ± 3.7

Education	King AbdulAziz University (17)	5 (3%)	11 (6.5%)	1 (0.6%)	0.939	8.5 ± 2.6
	King Saud University (12)	3 (1.8%)	9 (5.4%)	0		9.4 ± 2.4
	Taif University (65)	17 (10%)	47 (28%)	1 (0.6%)		9 ± 3
	Umm Al-Qura University (23)	5 (3%)	18 (10.7%)	0		9 ± 2.2
	Other (51)	1 (0.6%)	36 (21.5%)	2 (1.2%)		9.6 ± 2.7

Experience	Less than 1 year (26)	10 (6%)	16 (10%)	0	0.192	8.5 ± 2.6
	1–5 years (47)	8 (4.8%)	36 (21.5%)	3 (1.8%)		9.9 ± 2.3
	5–10 years (33)	10 (6%)	23 (13.7%)	0		8.2 ± 2.7
	More than 10 years (62)	15 (9%)	46 (27.5%)	1 (0.6%)		9.5 ± 3

Speciality	Pediatrics (18)	6 (3.6%)	11 (6.5%)	1 (0.6%)	0.028	9.5 ± 4
	Geriatrics (14)	1 (0.6%)	13 (7.7%)	0		10.8 ± 1.9
	Sport injuries (15)	7 (4.2%)	7 (4.2%)	1 (0.6%)		7.8 ± 2.2
	Long-term care units (11)	0	11 (6.5%)	0		10.5 ± 1.5
	Orthopedics (76)	15 (9%)	59 (35%)	2 (1.2%)		9.6 ± 2.5
	Others (43)	14 (8.3%)	20 (12%)	0		7.7 ± 2.9

^*∗*^OKAT = Osteoporosis Knowledge Assessment Tool categories (poor, good, and excellent). ^ƚ^*n* (%) = frequency and percentage from total.

## Data Availability

The data used to support the findings of this study are available from the corresponding author upon request.

## Additional Points

*Key Messages*. (i) The majority of physical therapy providers in Saudi Arabia have extensive knowledge in osteoporosis. (ii) Almost 80% of responders were unaware that there is significant bone loss in the ten years following the onset of menopause. (iii) There was a lack of knowledge for the practice and prevention questions among physical therapy providers in Saudi Arabia.
